# DNA Methylation Patterns and Transcriptomic Data Were Integrated to Investigate Candidate Genes Influencing Reproductive Traits in Ovarian Tissue from Sichuan White Geese

**DOI:** 10.3390/ijms26073408

**Published:** 2025-04-05

**Authors:** Lin Ma, Xianzhi Zhao, Haiwei Wang, Zhuping Chen, Keshan Zhang, Jiajia Xue, Yi Luo, Hanyu Liu, Xinshuai Jiang, Jiayue Wang, Xiaohui Ma, Fanglei Mao, Yuhan Zhong, Yueyang Liu, Rui Deng, Yanli Zhou, Chao Wang, Youhui Xie, Ying Chen, Qigui Wang, Guangliang Gao

**Affiliations:** 1 Chongqing Engineering Research Center of Goose Genetic Improvement, Institute of Poultry Science, Chongqing Academy of Animal Science, Chongqing 402460, China; 17783415478@163.com (L.M.); zhaoxianzhi2002@163.com (X.Z.); wahwe@163.com (H.W.); 2019102008@stu.sicau.edu.cn (Z.C.); zhangkshlk1988@163.com (K.Z.); xuejiajia25@163.com (J.X.); homleestar@163.com (Y.L.); wangccq@foxmail.com (C.W.); xyh917111@163.com (Y.X.); chenying.cq@163.com (Y.C.); 2 College of Animal Sciences and Technology, Northeast Agricultural University, Harbin 150030, China; s230501039@neau.edu.cn (H.L.); s230501037@neau.edu.cn (X.J.); 3 College of Animal Science and Technology, Southwest University, Chongqing 402460, China; 13835215470@139.com (J.W.); 19158415146@163.com (X.M.); mfl0507@163.com (F.M.); 13709469872@139.com (Y.Z.); liuyueyang0110@hotmail.com (Y.L.); dr17602307930@outlook.com (R.D.); mmmml13@outlook.com (Y.Z.)

**Keywords:** DNA methylation, gene expression, reproductive traits, ovary, Sichuan white goose

## Abstract

Ovarian tissue is critical for goose reproduction. This study aimed to investigate gene regulation by DNA methylation in relation to the reproductive traits of geese. We performed whole-genome bisulfite sequencing (WGBS) on ovarian tissues from Sichuan white geese (high-laying-rate group: HLRG, ♀ = 3; low-laying-rate group: LLRG, ♀ = 3) during the laying period. The results showed a higher level of hypermethylated differentially methylated regions (DMRs) in the HLRG, indicating a higher overall methylation level compared to the LLRG. In total, we identified 2831 DMRs and 733 differentially methylated genes (DMGs), including 363 genes with upregulated methylation. These DMGs were significantly enriched in pathways related to microtubule function (GO:0005874; GO:0000226), GnRH secretion, thyroid hormone signaling, ECM-receptor interaction, and PI3K–Akt signaling. Integration with RNA-seq data identified eight overlapping genes between DMGs and differentially expressed genes (DEGs), with five genes (*CUL9*, *MEGF6*, *EML6*, *SYNE2*, *AK1BA*) exhibiting a correlation between hypomethylation and high expression. *EML6*, in particular, emerged as a promising candidate, potentially regulating follicle growth and development in Sichuan white geese. Future studies should focus on further verifying the role of the *EML6* gene. In conclusion, this study provides important insights into the regulatory mechanisms of DNA methylation influencing reproductive traits in geese, offering novel candidate markers for future goose breeding programs.

## 1. Introduction

Fertilization rate and hatching rate are recognized as critical components impacting the reproductive performance of Sichuan white geese [1]. Compared to *Anser cygnoides* and *Anser anser*, Sichuan white geese (*Anser cygnoides*) exhibit the highest egg production rate (41.67%) and fertilization rate (96.92%), with successful mating occurring an average of 2.31 times per day [2]. The reproductive performance of laying female geese is influenced by various factors, including diet, nutrition, hormonal regulation, and environmental conditions [3,4,5]. Among these, hormonal changes play a critical role [6]. For example, elevated estrogen levels in female geese are strongly associated with ovarian development and ovulation cycles [7]. Hunzicker-Dunn et al. demonstrated that gonadotropins stimulate the release of follicle-stimulating hormone (FSH) and luteinizing hormone (LH), thereby regulating ovarian reproductive cycle initiation and impacting follicle development and subsequent ovulation [8]. Furthermore, Yang et al. found that progesterone (PROG) secretion can affect the development of stratified follicles and ovulation rate in laying geese [9].

The maturity of molecular genetic marker technology has made livestock and poultry quantitative trait mapping more robust and comprehensive, providing new means for livestock and poultry improvement. DNA methylation, a widespread epigenetic modification found in mammals, birds, reptiles, and insects, primarily occurs in promoter and genomic regions, exerting regulatory control over gene expression [10,11,12,13]. The primary mechanism involves the addition of methyl groups to cytosine bases, particularly within CpG islands, which typically leads to gene silencing [14,15]. Methylation contributes to a more compact chromatin structure by hindering transcription factor binding to promoter regions or by recruiting inhibitory proteins, such as methyl-CpG binding proteins (MeCPs), thereby impeding the assembly of the transcription machinery [16]. Beyond gene regulation, DNA methylation plays a critical role in maintaining genome stability by suppressing the activity of transposons and repetitive elements [17,18]. Methylation modifications, by altering gene expression patterns, influence key cellular processes, including differentiation, development, and environmental adaptation [19].

In recent years, significant progress has been made in studying the important economic traits of animals through whole-genome methylation analysis. Whole-genome methylation sequencing has been used to map the methylation profiles of key economic traits in animals, such as the regulation of DNA methylation on meat quality traits in cattle, pigs, and other domestic animals. Zhao and colleagues characterized the methylation profiles related to meat tenderness and toughness in Angus beef cattle and identified *UHRF1*, *NAALAD2*, *PLA2G4A,* and *ANTXR1* as candidate marker genes for DNA methylation regulating beef tenderness [20]. Luo et al. [21] conducted genome-wide association studies (GWASs) in muscle tail-fat tissues of DairyMeade sheep (DM3M) and Mongolian sheep (MS) and found that the *CAMK2D* gene in the DMRs may play a key role in fat metabolism and meat quality traits. Wang et al. [22] found, from DNA methylation data of muscle tissue of Chinese Chenghua (CHP) and Yorkshire pigs (YP), that the difference in flesh color between CHP and YP was the high level of methylation of the *ADCY1* and *PIK3R1* genes, which then affects the outcome of the change in its expression. These results help us understand how DNA methylation affects the expression of genes related to meat quality traits. At the same time, it also provides ideas for further exploring the epigenetic regulatory mechanisms of poultry DNA methylation affecting important economic traits.

We are particularly interested in investigating the role of DNA methylation in regulating prolificacy characteristics in poultry, with a specific focus on reproductive traits, such as egg production and egg production rate. Zhu et al. [23] further investigated the molecular mechanisms of chicken follicle development and ovulation, identifying genes encoding key enzymes involved in progesterone synthesis (including *Star*, *Cyp11a1*, and *Hsd3b*). Sagvekar et al. [24] demonstrated that the DNA methylation analysis of cumulus granulosa cells revealed methylation changes in the *CASR* and *TNF* genes, which regulate ovarian oocyte development and ovulation in polycystic ovary syndrome (PCOS). Chen et al. [25] compared and analyzed ovarian follicle data from hens with a high laying rate (HR) and low laying rate (LR) in three developmental stages, exploring the methylation landscape related to egg production and identifying 18 candidate genes, including *P2RX1*, *CAB39L*, *BLK*, and *CSMD3*.

Building upon our previous work identifying differentially methylated genes in pituitary tissue [26], in this experiment, we employed Sichuan white geese in the laying period as a model to analyze the DNA methylation profile of ovarian tissue and construct a comprehensive ovarian tissue methylome. Integrated with RNA-seq data, we explored genome-wide DNA methylation modifications to identify key prolificacy genes involved in the regulation of DNA methylation. Our findings contribute to the understanding of the molecular mechanisms influencing prolificacy traits in geese, providing novel epigenetic markers for molecular breeding and marker-assisted selection. In addition, this study lays a foundation for breeding high-laying-rate goose breeds and significantly promotes the advancement of poultry genetics.

## 2. Results

### 2.1. Egg Production Statistics

As can be seen in Table 1, compared with the low-laying-rate group (LLRG), total egg production (TEP) and monthly egg production (MEP) in the high-laying-rate group (HLRG) were significantly increased by 78.20% and 78.26%, respectively (*p* < 0.05). In addition, the egg production rate (LR) was significantly increased by 78.17% (*p* < 0.05).

### 2.2. Summary of RNA-Seq Sequencing Data

**Figure 1:**
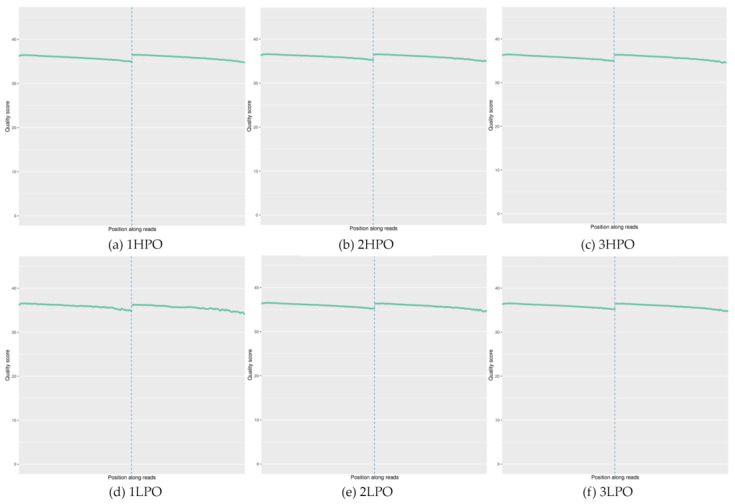
Sequencing quality distribution. Note: The horizontal coordinate is the base position of reads. The ordinate is the single base mass value. The first half is divided into the quality distribution of the first end of the double-ended sequencing reads. The second half is divided into the quality distribution of the other end of the sequencing reads. 1HPO to 3HPO represent the samples from the HLRG, and 1LPO to 3LPO represent the samples from the LLRG. HLRG: high-laying-rate group; LLRG: low-laying-rate group.

Ovarian tissue RNA sequencing was conducted to profile transcriptome differences between the high-laying-rate group (HLRG) and low-laying-rate group (LLRG) geese. Three individuals were randomly selected from each group (*n* = 3; N = 17 per group, respectively; Figure 1, Table 2). A total amount of 44.62 G of raw data was produced, and 42.54 G of clean data was filtered. The average Q20 and Q30 values for both groups were 97.44 and 93.27%, respectively, indicating sufficient sequencing quality for subsequent analysis.

### 2.3. Summary of Data from Genome-Wide Methylation Sequencing

**Figure 2:**
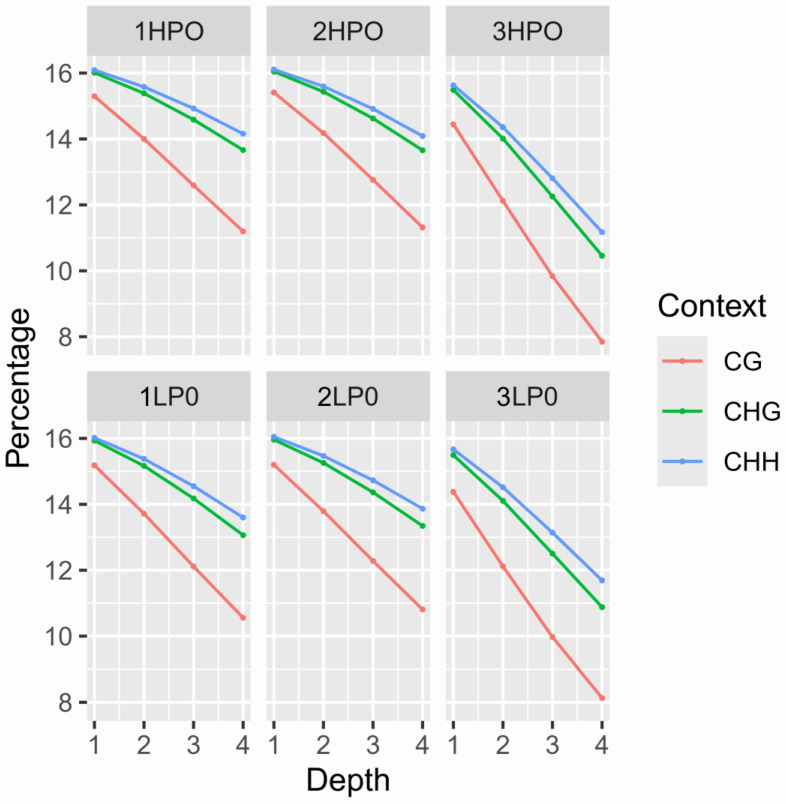
Different types of methylation sequencing depths. Note: 1HPO to 3HPO represent the samples from the HLRG, and 1LPO to 3LPO represent the samples from the LLRG. HLRG: high-laying-rate group; LLRG: low-laying-rate group.

To identify differences in DNA methylation patterns, whole-genome bisulfite sequencing (WGBS) was performed on ovarian tissue from three randomly selected female geese in both the high-laying-rate group (HLRG, n = 3) and low-laying-rate group (LLRG, n = 3) (N = 17 per group; Table 3). The HLRG and LLRG generated 19.55 and 18.63 Gb of raw sequence data, respectively. After data filtering, 15.61 and 15.37 Gb of clean reads were obtained, achieving a unique mapping rate of 79.10%. The average bisulfite conversion efficiency for both groups was 99.90%, indicating high-quality data with a high conversion rate (Table 3). Figure 2 shows the methylation sequencing depth of each sample at different types, with a coverage of four times. This coverage was sufficient for subsequent methylation analysis.

### 2.4. Analysis of Different Types of Methylation Levels

In this study, genome-wide DNA methylation levels were compared between two groups of ovarian samples. Through genome-wide methylation sequencing, we identified three major methylation types: mCG, mCHG, and mCHH. In the high-yield group (HLRG), the number of mCG methylation sites was 145,307,448, accounting for 63.90% of the total, and in the low-yield group (LLRG), the number of mCG sites was 153,422,600, accounting for 63.86%. In addition, the number of mCHG and mCHH methylation sites was less than 2%, indicating that mCG was the dominant methylation type in ovarian tissue of Sichuan white geese (Table 4).

### 2.5. Analysis of Differential Methylation Regions (DMRs)

**Figure 3:**
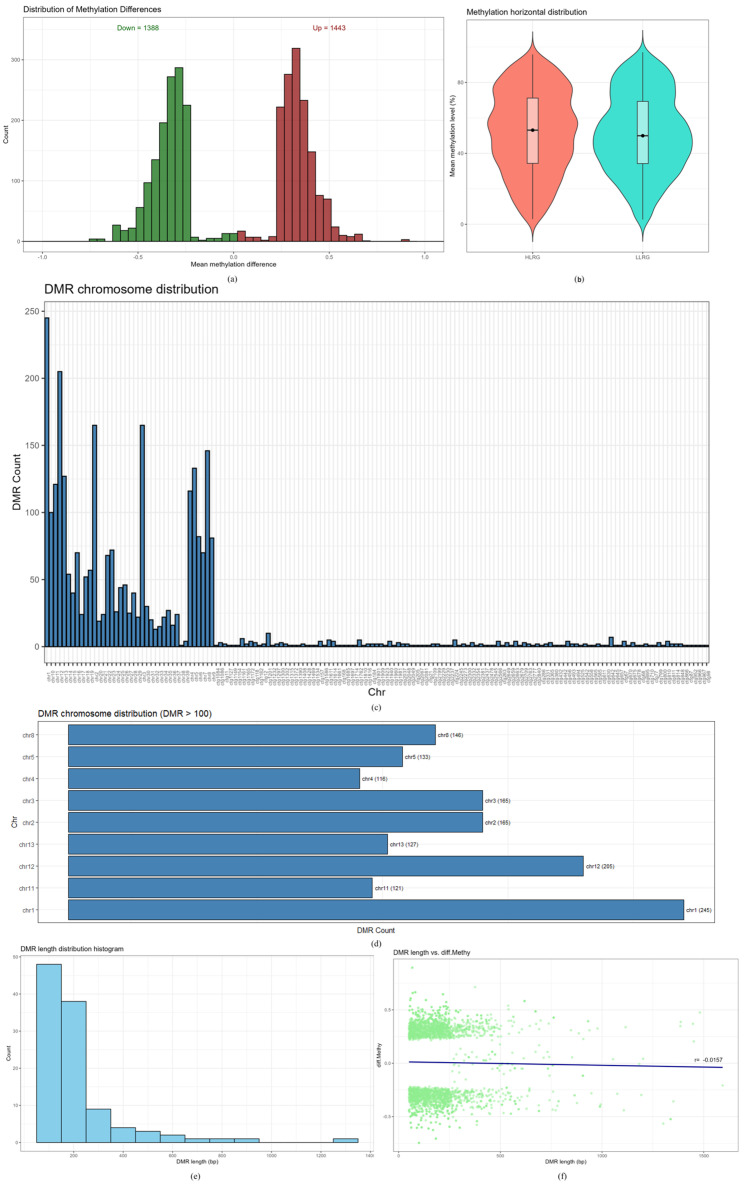
Basic descriptive statistical analysis of differentially methylated regions (DMRs). Note: (**a**): Number of DMRs between high-laying-rate group (HLRG) and low-laying-rate group (LLRG). (**b**): Diagram of mean methylation level of DMRs. (**c**): Histogram of DMRs distribution on each chromosome. (**d**): Bar chart with more than 100 DMRs. (**e**): Histogram of DMR length. (**f**): Scatter plot of relationship between DMR length and differential methylation levels.

DSS software (version 2.52.0) was used to identify differentially methylated regions (DMRs). A total of 2831 DMRs were identified between the high-yield group (HLRG) and low-yield group (LLRG). Of these, 1388 DMRs were hypomethylated, and 1443 were hypermethylated, indicating that DMRs were mainly hypermethylated (Figure 3a and Supplementary Table S1). The mean methylation level of DMRs in the HLRG was higher compared to the LLRG (Figure 3b). The distribution of DMRs across chromosomes (Figure 3c,d) showed that nine chromosomes (1, 2, 3, 4, 5, 8, 11, 12, and 13) contained over 100 DMRs each, with chromosome 1 having the highest number (245). The histogram in Figure 3e illustrates the DMRs length distribution, which predominantly ranged between 50 and 300 base pairs (bps). A scatter plot analysis of DMRs length and differential methylation levels (Figure 3f) yielded a Pearson correlation coefficient (r) of −0.0157, indicating a negative correlation. Specifically, as DMRs length increased, the differential methylation levels tended to decrease.

### 2.6. Association Analysis of Differentially Methylated Genes (DMGs)

**Figure 4:**
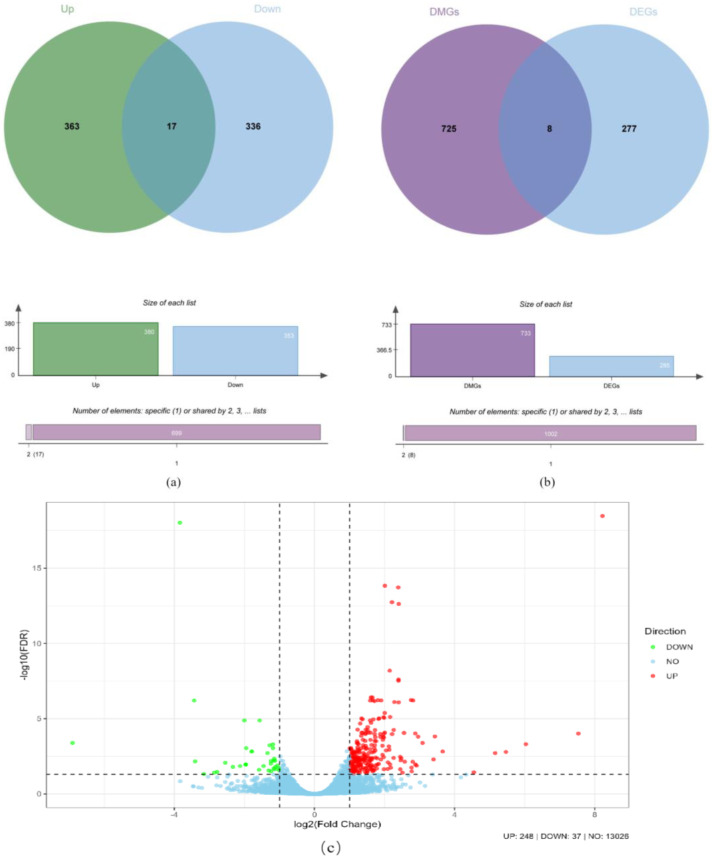
Co-analysis of differentially expressed genes (DEGs) and differentially methylated genes (DMGs). Note: (**a**): Distribution of up- and downregulated genes within DMGs (Venn diagram). (**b**): Venn diagram illustrating the overlap between DEGs and DMGs. (**c**): Volcano plot of DEGs.

Bedtools software (version 2.31.1) was used to identify differentially methylated genes (DMGs). From the differentially methylated regions (DMRs), a total of 733 DMGs were identified. Of these DMGs, 363 were associated with hypermethylated DMRs, 336 were associated with hypomethylated DMRs, and 17 were associated with both hypermethylated and hypomethylated DMRs (Figure 4a and Supplementary Table S2). In this study, differentially expressed genes (DEGs) were identified using padj < 0.05 and |log2 (FoldChange)| > 1. This analysis revealed a total of 285 DEGs between the high-yield group (HLRG) and low-yield group (LLRG), as shown in Figure 4c. Of these, 248 genes exhibited an increased expression, and 37 genes exhibited a decreased expression in ovarian tissue. Joint analysis of DMGs and DEGs revealed eight overlapping genes (Figure 4b, Table 5 and Supplementary Table S3). Among these, five genes (*CUL9*, *MEGF6*, *EML6*, *SYNE2*, and *AK1BA*) showed a correlation between hypomethylation and high expression. The remaining three genes (*MYCT1*, *F16B1*, and *TENA*) exhibited a high hypermethylation alongside high expression.

### 2.7. Enrichment and Network Interaction Analysis of Differentially Methylated Genes (DMGs)

**Figure 5:**
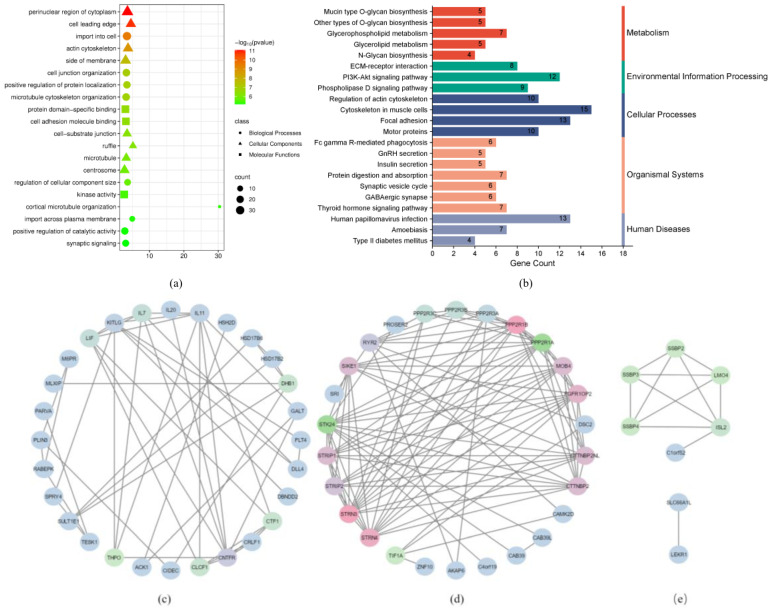
Differentially methylated genes (DMGs) enrichment and network interaction analysis. Note: (**a**): Gene Ontology (GO) enrichment analysis of DMGs. (**b**): Kyoto Encyclopedia of Genes and Genomes (KEGG) enrichment analysis of DMGs. (**c**–**e**): DMGs network interaction analysis, where the lines represent correlations between interacting genes.

Gene Ontology (GO) and Kyoto Encyclopedia of Genes and Genomes (KEGG) enrichment analyses were carried out for all the differentially methylated genes (DMGs) using the online version of Metascape (version 3.5) (https://metascape.org, accessed 25 July 2024). As shown in Figure 5a, GO enrichment analysis showed that DMGs in ovarian tissue were mainly enriched in cellular components, biological processes, and molecular function pathways. Microtubule function (GO:0005874, 23/486, *p* = 1.02 × 10^−6^), microtubule cytoskeleton organization (GO:0000226, 27/578, *p* = 1.51 × 10^−7^), centrosome (GO:0005813, 29/729, *p* = 1.41 × 10^−6^), and cortical microtubule organization (GO:0043622, 4/9, *p* = 5.37 × 10^−6^) are related to reproductive traits, such as cell division and material transport, in geese. KEGG pathway analysis (Figure 5b) indicated an enrichment in pathways, including GnRH secretion, thyroid hormone signaling, insulin secretion, ECM–receptor interaction, PI3K–Akt signaling, and actin cytoskeleton regulation, suggesting associations with reproductive traits, such as hormone regulation, ovulation, and follicle development. Protein–protein interaction network analysis (Figure 5c–e) identified 12 DMGs (*ACK1*, *LEKR1*, *DHB1*, *ISL2*, *TESK1*, *RYR2*, *PLIN3*, *KITLG*, *DLL4*, *CNTFR*, *TIF1A*, *STK24*) related to reproductive traits, such as gonadotropin secretion and the regulation of primordial follicle activation, with five DMGs (*ISL2*, *RYR2*, *KITLG*, *CNTFR*, and *STK24*) serving as key nodes.

## 3. Discussion

Improving reproductive traits is an important priority in goose breeding. Understanding the molecular mechanisms regulating these traits can accelerate breeding progress. In this study, we integrated whole-genome bisulfite sequencing (WGBS) and RNA-seq to compare DNA methylation patterns and gene expression profiles in the ovarian tissue of Sichuan white geese in the high-laying-rate group (HLRG) and low-laying-rate group (LLRG). The whole-genome methylation map of ovarian tissue from Sichuan white geese in the HHRG and LLRG revealed that CG methylation accounted for more than 63% of all methylated cytosines compared with the CHG and CHH sites, indicating that ovarian tissue methylation mainly occurred at the CG site. This was consistent with the methylation experiments by Zhao (*Wanxi white geese*) and Bao (Muscovy duck) [27,28]. This shows that the change in DNA methylation level is mainly to study methylation differences in CG sequence sites.

DNA methylation, an important epigenetic mechanism, is extensively used in plant research to analyze plant growth, development, stress resistance, and genome stability. For example, studies of 5mC modification in maize have shown that it regulates the expression of the key genes *ZmRap2.7* and *ZmNAC111*, affecting flowering time and drought resistance, offering new avenues for improving crop yield and resilience [29]. In our study, we focused on DNA methylation patterns at the CG sites in Sichuan white goose ovarian tissue and further identified a total of 2831 DMRs. Furthermore, we observed that the overall methylation level of DMRs in the HLRG was higher than that in the LLRG, suggesting a greater proportion of methylated cytosine bases in ovarian tissue DNA molecules from the HLRG group (high-laying geese).

In addition, a total of 733 differentially methylated genes (DMGs) were identified from these differentially methylated regions (DMRs), among which 363 genes had DMRs with upregulated methylation, 336 genes had DMRs with downregulated methylation, and 17 genes had two types of DMRs. This indicates that DMGs are mainly upregulated in methylation level. Furthermore, we found 12 genes (*ACK1*, *LEKR1*, *DHB1*, *ISL2*, *TESK1*, *RYR2*, *PLIN3*, *KITLG*, *DLL4*, *CNTFR*, *TIF1A*, *STK24*) from these DMGs that might be related to the regulation of reproductive traits in Sichuan white geese. In this study, the six DMGs (*ACK1*, *LEKR1*, *DHB1*, *RYR2*, *TIF1A*, *STK24*) showed hypermethylation. Sundaresan and Mota [30,31] found that the *RYR2* gene may be involved in gonadotropin-releasing hormone (GnRH) induced follicle stimulating hormone (FSH) and luteinizing hormone (LH) secretion. The *LEKR1*, *ACK1*, *TIF1A*, and *DHB1* genes are all implicated in ovarian follicle development during reproduction. Specifically, Mear [32] observed significant *LEKR1* expression in ovarian oocytes. Yan et al. [33] reported that *ACK1*, a member of the Rho GTPase family and an important cell regulator, is primarily found in oocytes, especially within activated follicles. Sun et al. [34] found that the *DHB1* gene, expressed in ovaries, encodes an enzyme involved in 17β-estradiol biosynthesis and plays a role in follicle development. Niederreither et al. [35] demonstrated that *TIF1A* regulates oocyte development and maturation in different stages of follicle development. Additionally, Lin et al. [36] showed that *STK24* maintains ovarian function. Interestingly, in our study, the other six DMGs (*ISL2*, *TESK1*, *PLIN3*, *KITLG*, *DLL4*, *CNTFR*) exhibited a hypomethylation state. These genes participate in the hypothalamic–pituitary–ovarian axis and play important roles in inducing ovulation [37,38], promoting oocyte growth and follicle development [39,40,41], and regulating the formation of blood vessels and tissue structures during follicle growth [42], among other reproductive processes.

To further verify whether these DMGs are related to reproductive trait pathways, we performed Gene Ontology (GO) and Kyoto Encyclopedia of Genes and Genomes (KEGG)enrichment analyses. GO enrichment analysis revealed a significant enrichment of DMGs in terms of microtubule function (GO:0005874), microtubule cytoskeleton organization (GO:0000226), centrosome (GO:0005813), and cortical microtubule organization (GO:0043622). Consistent with these findings, Albertini and colleagues [43] found that microtubule changes affect ovulation and oocyte maturation. Likewise, Zampolla et al. [44] also discussed the interaction between tubulin and mitochondria in granulosa cells, suggesting that mitochondria and tubulin were colocalized in stage III zebrafish ovarian follicles. These observations further support the reliability of our DMG results. In addition, KEGG pathway enrichment analysis revealed significant enrichment in pathways including GnRH secretion, thyroid hormone signaling, ECM–receptor interaction, and PI3K–Akt signaling. Supporting the involvement of GnRH secretion, Moenter and Vieyra-Valdez [45,46] reported its role in LH secretion from the anterior pituitary gland prior to ovulation as well as in the induction of ovulation itself. Notably, the RYR2 gene, identified as a DMG in our study, is also implicated in GnRH secretion, which aligns with this enrichment result. Wei [47] and Fedail [48] revealed that the thyroid hormone can modulate estrous cyclicity in the ovary, promote follicle development and maturation, and maintain various endocrine functions. Furthermore, ECM–receptor interaction is mainly related to follicular development, which is consistent with the study of Li [49]. Additionally, Long et al. [50] found that the PI3K–Akt signaling pathway affects follicle growth synergistically with progesterone and FSH. While these 12 DMGs related to reproductive traits did not overlap with the RNA-seq data in our study, further investigation of these genes could provide valuable insights into the genetic mechanisms underlying high-yielding traits in geese and provide a theoretical basis for future goose breeding efforts.

In this study, 285 differentially expressed genes (DEGs) were identified in ovarian tissue from Sichuan white geese. To identify genes exhibiting concurrent changes in both DNA methylation and mRNA expression, we combined WGBS and RNA-seq data, which revealed eight overlapping genes between the DMGs and DEGs. Among these, five genes (*CUL9*, *MEGF6*, *EML6*, *SYNE2*, and *AK1BA*) showed a correlation between hypomethylation and high expression. Three genes (*MYCT1*, *F16B1*, and *TENA*) exhibited a high methylation status alongside high expression. Yin [51] observed a high and preferential expression of *EML6*, a member of the *EML* protein family, in ovarian follicles, particularly within oocytes. Tian et al. [52] reported a correlation between the *SYNE2* gene and litter size in sheep, providing a marker-assisted selection site for high-fertility traits in sheep. Sell-Kubiak et al. [53] discussed the association between *CUL9* expression in embryos and pig litter size. The hypomethylation of *CUL9*, *EML6*, and *SYNE2* in ovarian tissue suggests their active role in reproductive processes, such as maintaining ovarian function and promoting follicle development, ultimately leading to their expression in ovarian tissue. Furthermore, Ozanne et al. [54] found that *TENA*, an extracellular matrix protein, is commonly used as a marker for inflammatory diseases in clinical medicine. Tshilate et al. [55] noted that the *MEGF6* gene contains an EGF-like domain, which functions in regulating cell proliferation and growth during tissue cell injury and repair. By analyzing the relationship between DMGs and DEGs, we have elucidated how these epigenetic modifications affect gene expression, thereby regulating reproductive traits in geese.

## 4. Materials and Methods

### 4.1. Experimental Animals and Sample Collection

This study was approved by the Experimental Animal Ethics Committee of Chongqing Academy of Animal Sciences. All Sichuan white geese involved in this study were from the Goose Breeding Center of Chongqing Academy of Zoology. During the experiment, Sichuan white geese were raised in a single cage at the Waterfowl Experimental base of Anfu County Waterfowl Breeding Base (105.48° N, 29.34° E), uniformly fed standard feed, immunized, and deinsected. A total of 230 Sichuan white geese were randomly selected to record their egg production from first birth to 45 weeks of age. According to egg production, individuals with egg production greater than 47 were divided into the high-laying-rate group(HLRG), individuals with egg production lower than 35 were divided into the low-laying-rate group (LLRG), and 34 Sichuan white geese (17 in the HLRG and 17 in theLLR duction (MEP), and laying rate (LR) [56], and the formula is as follows:

$$LR\;\left(\%\right)=\left(\frac{P}{M}\right)\times 100$$

where P stands for the total number of eggs produced during the trial period and M stands for test days.

In this study, to precisely determine the timing of egg laying, our goose house was equipped with a real-time Internet of Things system that continuously monitored prenatal behavior and egg-laying events. Prior to the formal experiment, we performed intensive longitudinal observation of the goose population, recording detailed laying times and behaviors for individual geese. Through the analysis of these historical data, the subsequent laying time of the six individuals selected for the formal experiment was accurately predicted. The experimental statistical sample size was based on Cunningham et al. [57]. Approximately 2 h prior to predicted oviposition, 6 Sichuan white geese were randomly selected from the HLRG (n = 3) and LLRG (n = 3) for euthanasia; complete ovarian tissue (including cortex and medulla) was rapidly dissected; and extraneous blood and connective tissue were removed, divided into cryogenic tubes, immediately frozen in liquid nitrogen, and transferred to −80 °C for storage. Tissue homogenates were then prepared using a tissue homogenizer for subsequent WGBS and transcriptome analysis.

### 4.2. Total RNA Extraction, Library Preparation, and Sequencing

Total RNA was extracted using Trizol reagents (Invitrogen, Carlsbad, CA, USA) according to the manufacturer’s protocol. A NanoDrop ND-1000 (NanoDrop Technologies, Wilmington, DE, USA) was used to detect the purity of the RNA at an OD ratio of 260/280. The integrity of the RNA was accurately detected using an Agilent 2100 Bioanalyzer (Agilent Technologies, Santa Clara, CA, USA), and the concentration of RNA was accurately quantified using Qubit 2.0. RNA contamination and degradation were evaluated using 1% agarose gel electrophoresis. Then, 3 μg of total RNA was extracted from each ovarian tissue sample to enrich mRNA by magnetic beads with Oligo (dT) and cleaved into fragments of about 150 bp. cDNA libraries were constructed using these fragments, and then, the cDNA library was utilized by the Illumina NovaSeq 6000 platform (Illumina, San Diego, CA, USA) for sequencing. Quality control was performed on the sequencing data, and low-quality data of raw reads and ploy-N were deleted to obtain clean reads. The mapped, CR, Q30, and GC contents of clean reads were calculated [58].

### 4.3. DNA Extraction and Whole-Genome Bisulfite Sequencing (WGBS) Library Construction

Genomic DNA was extracted from the rapid-frozen ovarian tissue using the QIAamp DNA mini-Kit (Qiagen, NRW, Hilden, Germany) for each group of ovarian tissue samples according to the recommended instructions. The DNA was randomly fragmented to 200–300 bp using ultrasound. Then, end repair was performed, 3 ’plus A base and a joint. The bisulfite treatment of DNA converts the unmethylated cytosine into uracil, while the methylated cytosine remains unchanged. Subsequently, the resulting libraries were subjected to paired-end sequencing with a 150 bp read length using the Illumina HiSeq™ 2500 platform (Illumina, San Diego, CA, USA) of Beijing Nova Genomics Co., Ltd., Beijing, China.

### 4.4. Whole-Genome Bisulfite Sequencing (WGBS) Data Processing

To ensure the quality of subsequent analysis, raw sequencing reads were first assessed for quality using FastQC (version 0.11.5) and trimmed with Trimmomatic (version 0.39) to remove low-quality sequences and obtain high-quality reads. The goose reference genome [59] (NCBI accession ASM1303099) was retrieved from the NCBI database (https://www.ncbi.nlm.nih.gov/, accessed 13 October 2024). To facilitate alignment, both sequenced reads and the reference genome were converted to their bisulfite-treated counterparts, substituting C-to-T and G-to-A. The converted reference genome was indexed using Bowtie2 (version 2.2.9) to enhance alignment efficiency, and the comparison scoring threshold was set as score_minL,0,−0.6. In the comparison results, reads aligning to the same genomic region were considered duplicates. Sequencing depth and coverage were determined by the number of duplicate reads. Bismark (version 0.23.0) was used to generate methylation information in different backgrounds (CG, CHG, CHH). The methylation level was defined as the number of methylated C bases divided by the sum of the number of methylated C bases and the number of unmethylated C bases. The sequencing data from ovarian tissues of Sichuan white geese have been deposited in the NCBI Sequence Read Archive (SRA).

### 4.5. Differential Methylation Regions (DMRs) Analysis

Differential Methylation Regions (DMRs) were identified using DSS (version 2.52.0) software, employing a Bayesian hierarchical model-based shrinkage estimator for discrete parameter estimation. Analysis was performed using a Wald test based on the β-binomial distribution, incorporating discrete parameter characterization and sequencing depth. First, differentially methylated genes (DMGs) were identified by locating DMRs within gene boundaries based on their genomic position. DMRs were defined as the presence of at least one methylation site in the region, with a methylation level difference exceeding 25% and a *p*-value less than 0.05. To identify DMGs within these DMRs, DMGs were detected within selected genes using a *p*-value cut-off of < 0.05. Venn diagrams, generated using the online tool (https://www.bioinformatics.org/, accessed 20 July 2024), were used to visualize the overlap between gene sets.

### 4.6. Gene Ontology (GO) and Kyoto Encyclopedia of Genes and Genomes (KEGG) Enrichment Analysis

Genes associated with Differential Methylation Regions (DMRs) were annotated using the Ensembl database. A promoter region was defined as the 2 kb region upstream of the transcription start site (TSS), while the genomic region encompassed the TSS to the ATG start codon, coding sequence, and TTS. Genes annotated from DMRs (*p* < 0.05, methylation difference ≥ 0.25) were included in the enrichment analysis. GO enrichment analysis and the visualization of differentially methylated genes (DMGs) were performed using Metascape (version 3.5) (https://metascape.org/gp/index.html#/main/step1, accessed 25 July 2024). KEGG pathway analysis and the visualization of DMGs were conducted using the Wei Sheng Xin online tool (https://www.bioinformatics.com.cn/, accessed 28 August 2024). The STRING database (http://string-db.org/, accessed 5 August 2024) was utilized to analyze the interaction network associated with the reproductive pathway of selected DMGs.

### 4.7. Data Statistics

All data were analyzed using the R 4.3.1 software (R, Vienna, Austria). The high-laying-rate group (HLRG) and low-laying-rate group (LLRG) were compared using the independent-samples *t*-test. The results are expressed as the mean ± standard error of mean (SEM). Differences were considered significant at *p* < 0.05.

## 5. Conclusions

DNA methylation analysis of ovarian tissue in the high-laying-rate group (HLRG) and low-laying-rate group (LLRG) Sichuan white geese identified 2831 Differential Methylation Regions (DMRs) and 733 differentially methylated genes (DMGs). These DMGs were significantly enriched in reproductive pathways involved in hormone regulation, follicle growth, and oocyte development. Further analysis of RNA-seq data revealed eight overlapping genes, with the *EML6* candidate gene potentially associated with ovarian follicle growth and development in Sichuan white geese. Future research should validate the specific regulatory role of *EML6* within the ovary. Overall, this study elucidated the DNA methylation pattern of the Sichuan white goose ovary and explored the regulatory roles of DMGs and differentially expressed genes (DEGs) in the reproductive process, providing a new method to further promote the breeding of high-laying geese.

## Figures and Tables

**Table 1:** Summary of egg production data.

Items	HLRG	LLRG	*p*-Value
Sample	17	17	
TEP	55.76 ± 1.61	31.29 ± 1.23	<0.001
MEP	13.94 ± 0.40	7.82 ± 0.31	<0.001
LR/%	46.86 ± 1.35	26.30 ± 1.03	<0.001

Note: HLRG: high-laying-rate group; LLRG: low-laying-rate group; TEP: total egg production; MEP: monthly egg production; LR: egg production rate.

**Table 2:** RNA-seq sequencing data.

Groups	Sample	Raw Reads	Clean Reads	RB (G)	CB (G)	ER (%)	Q20 (%)	Q30 (%)
HLRG	1HPO	21,006,508	19,754,481	6.30	5.93	94.04	97.38	93.12
	2HPO	21,321,613	20,127,809	6.40	6.04	94.40	97.72	94.24
	3HPO	25,219,828	23,791,538	7.57	7.14	94.34	97.24	92.77
LLRG	1LPO	33,732,858	32,942,426	10.12	9.88	97.66	97.19	92.75
	2LPO	21,102,732	20,168,463	6.33	6.05	95.57	97.50	93.23
	3LPO	26,338,862	24,998,942	7.90	7.50	94.91	97.59	93.53

Note: HLRG: high-laying-rate group; LLRG: low-laying-rate group; RB: raw base; CB: clean base; ER: effective rate. 1HPO to 3HPO represent the samples from the HLRG, and 1LPO to 3LPO represent the samples from the LLRG. Q20: The percentage of bases with Phred values greater than 20 in the total base; Q30: the percentage of bases with Phred values greater than 30 in the total base.

**Table 3:** Genome-wide methylation sequencing data.

Groups	Sample	RR (Gb)	CB (Gb)	Clean Ratio (%)	Mapping rate (%)	BCR (%)
HLRG	1HPO	20.18	15.45	76.57	74.60	99.90
	2HPO	18.16	15.16	83.44	81.40	99.90
	3HPO	20.30	16.21	79.85	77.80	99.90
LLRG	1LPO	18.25	14.70	80.56	78.50	99.90
	2LPO	18.97	15.53	81.85	79.90	99.90
	3LPO	18.66	15.87	85.08	82.40	99.89

Note: HLRG: high-yield group; LLRG: low-yield group; 1HPO to 3HPO represent the samples from the HLRG, and 1LPO to 3LPO represent the samples from the LLRG. RR: raw read; CB: clean read; BCR: bisulfite conversion rate.

**Table 4:** Methylation levels of different types.

Groups	HLRG	LLRG
mCG (%)	145,307,448 (63.90%)	153,422,600 (63.86%)
mCHG (%)	706,246,411 (0.92%)	750,555,445 (0.99%)
mCHH (%)	2,233,876,832 (0.97%)	2,393,932,911 (1.06%)

Note: HLRG: high-yield group; LLRG: low-yield group. 1HPO to 3HPO represent the samples from the HLRG. mCpG: total methylated Cs in CpG context; mCHG: total methylated Cs in CHG context; mCHH: total methylated Cs in CHH context.

**Table 5:** Statistics of 8 overlapping genes between differentially expressed genes (DEGs) and differentially methylated genes (DMGs) in Venn diagram.

Gene	MDL	FC	*p*-Value	Padj	LR
*CUL9*	−0.31	1.95	1.93 × 10^−5^	1.75 × 10^−3^	21.78
*MEGF6*	−0.31	1.65	3.59 × 10^−4^	1.57 × 10^−2^	18.81
*EMAL6*	−0.27	1.07	8.84 × 10^−4^	3.61 × 10^−2^	11.06
*SYNE2*	−0.44	1.51	6.69 × 10^−4^	2.91 × 10^−2^	11.57
*AK1BA*	−0.28	1.59	7.46 × 10^−6^	1.06 × 10^−3^	20.07
*MYCT1*	0.38	1.72	4.37 × 10^−5^	3.28 × 10^−3^	22.21
*F16B1*	0.34	1.49	3.97 × 10^−5^	3.27 × 10^−3^	18.21
*TENA*	0.45	1.73	1.77 × 10^−7^	5.74 × 10^−5^	27.27

Note: DML: differential methylation level (HLRG vs. LLRG); FC: log2 (FoldChange) (HLRG vs. LLRG); *CUL9*: Cullin-9; *MEGF6*: Multiple epidermal growth factor-like domains protein 6; *EML6*: Echinoderm microtubule-associated protein-like 6; *SYNE2*: Nesprin-2; *AK1BA*: Aldo-keto reductase family 1 member B10; *MYCT1*: Myc target protein 1 homolog; *F16B1*: Protein FAM160B1; TENA: Tenascin. HLRG: high-yield group; LLRG: low-yield group.

## Data Availability

The original contributions presented in this study are included in the article. Further inquiries can be directed to the corresponding author(s).

## Supplementary Materials

The following supporting information can be downloaded at: https://www.mdpi.com/article/10.3390/ijms26073408/s1.
